# Insights into ancestral diversity in Parkinson’s disease risk: a comparative assessment of polygenic risk scores

**DOI:** 10.1038/s41531-025-00967-4

**Published:** 2025-07-03

**Authors:** Paula Saffie-Awad, Spencer M. Grant, Mary B. Makarious, Inas Elsayed, Arinola O. Sanyaolu, Peter Wild Crea, Artur F. Schumacher Schuh, Kristin S. Levine, Dan Vitale, Mathew J. Koretsky, Jeffrey Kim, Thiago Peixoto Leal, María Teresa Periñán, Sumit Dey, Alastair J. Noyce, Armando Reyes-Palomares, Noela Rodriguez-Losada, Jia Nee Foo, Wael Mohamed, Karl Heilbron, Lucy Norcliffe-Kaufmann, the 23andMe Research Team, Stella Aslibekyan, Adam Auton, Elizabeth Babalola, Robert K. Bell, Jessica Bielenberg, Katarzyna Bryc, Emily Bullis, Paul Cannon, Daniella Coker, Gabriel Cuellar Partida, Devika Dhamija, Sayantan Das, Sarah L. Elson, Nicholas Eriksson, Teresa Filshtein, Alison Fitch, Kipper Fletez-Brant, Pierre Fontanillas, Will Freyman, Julie M. Granka, Alejandro Hernandez, Barry Hicks, David A. Hinds, Ethan M. Jewett, Yunxuan Jiang, Katelyn Kukar, Alan Kwong, Keng-Han Lin, Bianca A. Llamas, Maya Lowe, Jey C. McCreight, Matthew H. McIntyre, Steven J. Micheletti, Meghan E. Moreno, Priyanka Nandakumar, Dominique T. Nguyen, Elizabeth S. Noblin, Jared O’Connell, Aaron A. Petrakovitz, G. David Poznik, Alexandra Reynoso, Madeleine Schloetter, Morgan Schumacher, Anjali J. Shastri, Janie F. Shelton, Jingchunzi Shi, Suyash Shringarpure, Qiaojuan Jane Su, Susana A. Tat, Christophe Toukam Tchakouté, Vinh Tran, Joyce Y. Tung, Xin Wang, Wei Wang, Catherine H. Weldon, Peter Wilton, Corinna D. Wong, Mie Rizig, Njideka Okubadejo, Mike A. Nalls, Cornelis Blauwendraat, Andrew Singleton, Hampton Leonard, Global Parkinson’s Genetics Program (GP2), Mie Rizig, Njideka Okubadejo, Mike A. Nalls, Cornelis Blauwendraat, Andrew Singleton, Hampton Leonard, Emilia M. Gatto, Marcelo Kauffman, Samson Khachatryan, Zaruhi Tavadyan, Claire E. Shepherd, Julie Hunter, Kishore Kumar, Melina Ellis, Miguel E. Rentería, Sulev Koks, Alexander Zimprich, Carlos Rieder, Vitor Tumas, Sarah Camargos, Edward A. Fon, Oury Monchi, Ted Fon, Benjamin Pizarro Galleguillos, Marcelo Miranda, Maria Leonor Bustamante, Patricio Olguin, Pedro Chana, Beisha Tang, Huifang Shang, Jifeng Guo, Piu Chan, Wei Luo, Gonzalo Arboleda, Jorge Orozco, Marlene Jimenez del Rio, Alvaro Hernandez, Mohamed Salama, Walaa A. Kamel, Yared Z. Zewde, Alexis Brice, Jean-Christophe Corvol, Ana Westenberger, Anastasia Illarionova, Brit Mollenhauer, Christine Klein, Eva-Juliane Vollstedt, Franziska Hopfner, Günter Höglinger, Harutyun Madoev, Joanne Trinh, Johanna Junker, Katja Lohmann, Lara M. Lange, Manu Sharma, Sergio Groppa, Thomas Gasser, Zih-Hua Fang, Albert Akpalu, Georgia Xiromerisiou, Georgios Hadjigeorgiou, Ioannis Dagklis, Ioannis Tarnanas, Leonidas Stefanis, Maria Stamelou, Efthymios Dardiotis, Alex Medina, Germaine Hiu-Fai Chan, Nancy Ip, Nelson Yuk-Fai Cheung, Phillip Chan, Xiaopu Zhou, Asha Kishore, K. P. Divya, Pramod Pal, Prashanth Lingappa Kukkle, Roopa Rajan, Rupam Borgohain, Mehri Salari, Andrea Quattrone, Enza Maria Valente, Lucilla Parnetti, Micol Avenali, Tommaso Schirinzi, Manabu Funayama, Nobutaka Hattori, Tomotaka Shiraishi, Altynay Karimova, Gulnaz Kaishibayeva, Cholpon Shambetova, Rejko Krüger, Ai Huey Tan, Azlina Ahmad-Annuar, Mohamed Ibrahim Norlinah, Nor Azian Abdul Murad, Shahrul Azmin, Shen-Yang Lim, Yi Wen Tay, Daniel Martinez-Ramirez, Mayela Rodriguez-Violante, Paula Reyes-Pérez, Bayasgalan Tserensodnom, Rajeev Ojha, Tim J. Anderson, Toni L. Pitcher, Oluwadamilola Ojo, Jan O. Aasly, Lasse Pihlstrøm, Manuela Tan, Shoaib Ur-Rehman, Mario Cornejo-Olivas, Maria Leila Doquenia, Raymond Rosales, Angel Vinuela, Elena Iakovenko, Bashayer Al Mubarak, Muhammad Umair, Eng-King Tan, Ferzana Amod, Jonathan Carr, Soraya Bardien, Beomseok Jeon, Yun Joong Kim, Esther Cubo, Ignacio Alvarez, Janet Hoenicka, Katrin Beyer, Pau Pastor, Sarah El-Sadig, Christiane Zweier, Paul Krack, Chin-Hsien Lin, Hsiu-Chuan Wu, Pin-Jui Kung, Ruey-Meei Wu, Serena Wu, Yih-Ru Wu, Rim Amouri, Samia Ben Sassi, A. Nazl Başak, Gencer Genc, Özgür Öztop Çakmak, Sibel Ertan, Alejandro Martínez-Carrasco, Anette Schrag, Anthony Schapira, Camille Carroll, Claire Bale, Donald Grosset, Eleanor J. Stafford, Henry Houlden, Huw R. Morris, John Hardy, Kin Y. Mok, Nicholas Wood, Nigel Williams, Olaitan Okunoye, Patrick A. Lewis, Rauan Kaiyrzhanov, Rimona Weil, Seth Love, Simon Stott, Simona Jasaitye, Vida Obese, Alberto Espay, Alyssa O’Grady, Andrew K. Sobering, Bernadette Siddiqi, Bradford Casey, Brian Fiske, Cabell Jonas, Carlos Cruchaga, Caroline B. Pantazis, Charisse Comart, Claire Wegel, Deborah Hall, Dena Hernandez, Ejaz Shamim, Ekemini Riley, Faraz Faghri, Geidy E. Serrano, Hirotaka Iwaki, Honglei Chen, Ignacio Juan Keller Sarmiento, Jared Williamson, Joseph Jankovic, Joshua Shulman, Justin C. Solle, Kaileigh Murphy, Karen Nuytemans, Karl Kieburtz, Katerina Markopoulou, Kenneth Marek, Lana M. Chahine, Laurel Screven, Lauren Ruffrage, Lisa Shulman, Luca Marsili, Maggie Kuhl, Marissa Dean, Miguel Inca-Martinez, Naomi Louie, Niccolò E. Mencacci, Roger Albin, Roy Alcalay, Ruth Walker, Sohini Chowdhury, Sonya Dumanis, Steven Lubbe, Tao Xie, Tatiana Foroud, Thomas Beach, Todd Sherer, Yeajin Song, Duan Nguyen, Toan Nguyen, Masharip Atadzhanov, Ignacio F. Mata, Sara Bandres-Ciga, Ignacio F. Mata, Sara Bandres-Ciga

**Affiliations:** 1 Programa de Pós-Graduação em Ciências Médicas, Universidade Federal do Rio Grande do Sul, Porto Alegre, Brazil; 2 Centro de Trastornos del Movimiento (CETRAM), Santiago, Chile; 3 Clínica Santa María, Santiago, Chile; 4 Centre for Alzheimer’s and Related Dementias. National Institute on Aging, National Institutes of Health, Bethesda, MD USA; 5 Laboratory of Neurogenetics, National Institute on Aging, National Institutes of Health, Bethesda, MD USA; 6 Department of Neuromuscular Diseases, UCL Queen Square Institute of Neurology, London, UK; 7 Faculty of Pharmacy, University of Gezira, Wadmadani, Sudan; 8 Department of Anatomy, College of Medicine, University of Lagos, Lagos, Nigeria; 9 Serviço de Neurologia, Hospital de Clínicas de Porto Alegre, Porto Alegre, Brazil; 10 Departamento de Farmacologia, Universidade Federal do Rio Grande do Sul, Porto Alegre, Brazil; 11 DataTecnica LLC, Washington, DC USA; 12 Genomic Medicine, Lerner Research Institute, Cleveland Clinic Foundation, Cleveland, OH USA; 13 Unidad de Trastornos del Movimiento, Servicio de Neurología y Neurofisiología Clínica, Instituto de Biomedicina de Sevilla, Hospital Universitario Virgen del Rocío/CSIC/Universidad de Sevilla, Seville, Spain; 14 Centro de Investigación Biomédica en Red sobre Enfermedades Neurodegenerativas (CIBERNED), Madrid, Spain; 15 Preventive Neurology Unit, Wolfson Institute of Population Health, Queen Mary University of London, London, UK; 16 Department of Molecular Biology and Biochemistry, Faculty of Sciences, University of Málaga, Málaga, Spain; 17 Faculty of Education Sciences, University of Málaga, Málaga, Spain; 18 Lee Kong Chian School of Medicine, Nanyang Technological University Singapore, Singapore, Singapore; 19 Laboratory of Neurogenetics, Genome Institute of Singapore, A*STAR, Singapore, Singapore; 20 Neuroscience Unit, Clinical Pharmacology Dept, Menoufia Medical School, Shebeen El-Kom, Egypt; 21 23andMe Inc., Sunnyvale, CA USA; 22 Department of Medicine, College of Medicine, University of Lagos, Lagos, Nigeria; 23 German Center for Neurodegenerative Diseases (DZNE), Tübingen, Germany; 24 Sanatorio de la Trinidad Mitre-INEBA, Buenos Aires, Argentina; 25 Hospital JM Ramos Mejia, Buenos Aires, Argentina; 26 Somnus Neurology Clinic, Yerevan, Armenia; 27 Neuroscience Research Australia, Sydney, NSW Australia; 28 ANZAC Research Institute, Concord, NSW Australia; 29 Garvan Institute of Medical Research and Concord Repatriation General Hospital, Darlinghurst, NSW Australia; 30 Concord Hospital, Concord, NSW Australia; 31 QIMR Berghofer Medical Research Institute, Herston, QLD Australia; 32 Murdoch University, Perth, WA Australia; 33 Medical University of Vienna, Vienna, Austria; 34 Universidade Federal do Rio Grande do Sul / Hospital de Clínicas de Porto Alegre, Porto Alegre, Brazil; 35 University of São Paulo, São Paulo, Brazil; 36 Universidade Federal de Minas Gerais, Belo Horizonte, Brazil; 37 McGill University, Montreal, QC Canada; 38 Universidad de Chile, Santiago, Chile; 39 Fundación Diagnosis, Santiago, Chile; 40 Faculty of Medicine, Universidad de Chile, Santiago, Chile; 41 CETRAM, Santiago, Chile; 42 Central South University, Changsha, China; 43 West China Hospital, Sichuan University, Chengdu, China; 44 Xiangya Hospital, Central South University, Changsha, China; 45 Capital Medical University, Beijing, China; 46 Zhejiang University, Hangzhou, China; 47 Universidad Nacional de Colombia, Bogotá, Colombia; 48 Fundación Valle del Lili, Santiago de Cali, Colombia; 49 University of Antioquia, Medellín, Colombia; 50 University of Costa Rica, San José, Costa Rica; 51 The American University in Cairo, Cairo, Egypt; 52 Beni-Suef University, Beni Suef, Egypt; 53 Addis Ababa University, Addis Ababa, Ethiopia; 54 Paris Brain Institute, Paris, France; 55 Sorbonne Université, Paris, France; 56 University of Lübeck, Lübeck, Germany; 57 Deutsches Zentrum für Neurodegenerative Erkrankungen (DZNE), Göttingen, Germany; 58 University Medical Center Göttingen, Göttingen, Germany; 59 University Hospital LMU Munich, Munich, Germany; 60 Hannover Medical School, Hannover, Germany; 61 University of Lübeck and University Medical Center Schleswig-Holstein, Lübeck, Germany; 62 University of Tübingen, Tübingen, Germany; 63 The German Center for Neurodegenerative Diseases (DZNE), Göttingen, Germany; 64 University of Ghana Medical School, Accra, Ghana; 65 University of Thessaly, Larissa, Greece; 66 Aristotle University of Thessaloniki, Thessaloniki, Greece; 67 Ionian University, Corfu, Greece; 68 Biomedical Research Foundation of the Academy of Athens, Athens, Greece; 69 Diagnostic and Therapeutic Centre HYGEIA Hospital, Marousi, Greece; 70 Hospital San Felipe, Tegucigalpa, Honduras; 71 Queen Elizabeth Hospital, Kowloon, Hong Kong; 72 The Hong Kong University of Science and Technology, Kowloon, Hong Kong; 73 Aster Medcity, Kochi, India; 74 Sree Chitra Tirunal Institute for Medical Sciences and Technology, Thiruvananthapuram, India; 75 National Institute of Mental Health & Neurosciences (NIMHANS), Bengaluru, India; 76 Manipal Hospital, Delhi, India; 77 All India Institute of Medical Sciences, Delhi, India; 78 Nizam’s Institute of Medical Sciences, Hyderabad, India; 79 Shahid Beheshti University of Medical Sciences, Tehran, Iran; 80 Magna Græcia University of Catanzaro, Catanzaro, Italy; 81 University of Pavia, Pavia, Italy; 82 University of Perugia, Perugia, Italy; 83 University of Rome Tor Vergata, Rome, Italy; 84 Juntendo University, Tokyo, Japan; 85 Juntendo University Faculty of Medicine, Tokyo, Japan; 86 Jikei University School of Medicine, Tokyo, Japan; 87 Institute of Neurology and Neurorehabilitation, Almaty, Kazakhstan; 88 Kyrgyz State Medical Academy, Bishkek, Kyrgyzstan; 89 Luxembourg Centre for Systems Biomedicine, University of Luxembourg, Belvaux, Luxembourg; 90 University of Malaya, Kuala Lumpur, Malaysia; 91 Universiti Kebangsaan Malaysia, Kuala Lumpur, Malaysia; 92 UKM Medical Molecular Biology Institute (UMBI), Kuala Lumpur, Malaysia; 93 Universiti Kebangsaan Malaysia Medical Centre, Kuala Lumpur, Malaysia; 94 Tecnológico de Monterrey, Monterrey, Mexico; 95 Instituto Nacional de Neurología y Neurocirugía, Mexico City, Mexico; 96 Universidad Nacional Autónoma de México, Mexico City, Mexico; 97 Mongolian National University of Medical Sciences, Ulaanbaatar, Mongolia; 98 Tribhuvan University, Kirtipur, Nepal; 99 University of Otago, Christchurch, New Zealand; 100 University of Otago, Dunedin, New Zealand; 101 College of Medicine, University of Lagos, Lagos, Nigeria; 102 Norwegian University of Science and Technology, Trondheim, Norway; 103 Oslo University Hospital, Oslo, Norway; 104 University of Science and Technology Bannu, Khyber Pakhtunkhwa, Pakistan; 105 Instituto Nacional de Ciencias Neurológicas, Lima, Peru; 106 Metropolitan Medical Center, Manila, Philippines; 107 University of Puerto Rico, San Juan, Puerto Rico; 108 Research Center of Neurology, Moscow, Russia; 109 King Faisal Specialist Hospital and Research Center, Riyadh, Saudi Arabia; 110 King Abdullah International Medical Research Center, Jeddah, Saudi Arabia; 111 National Neuroscience Institute, Singapore, Singapore; 112 University of KwaZulu-Natal, Durban, South Africa; 113 University of Stellenbosch, Stellenbosch, South Africa; 114 Stellenbosch University, Stellenbosch, South Africa; 115 Seoul National University Hospital, Seoul, South Korea; 116 Yongin Severance Hospital, Seoul, South Korea; 117 Hospital Universitario de Burgos, Burgos, Spain; 118 University Hospital Mutua Terrassa, Barcelona, Spain; 119 Institut de Recerca Sant Joan de Déu, Barcelona, Spain; 120 Research Institute Germans Trias i Pujol, Badalona, Spain; 121 University Hospital Germans Trias i Pujol, Badalona, Spain; 122 Faculty of Medicine, University of Khartoum, Khartoum, Sudan; 123 Inselspital, Bern University Hospital, University of Bern, Bern, Switzerland; 124 National Taiwan University Hospital, Taipei City, Taiwan; 125 Chang Gung Memorial Hospital, Taoyuan City, Taiwan; 126 National Taiwan University, Taipei City, Taiwan; 127 Chang Gung University, Taoyuan City, Taiwan; 128 National Institute Mongi Ben Hamida of Neurology, Tunis, Tunisia; 129 Mongi Ben Hmida National Institute of Neurology, Tunis, Tunisia; 130 Koç University, Istanbul, Turkey; 131 Şişli Etfal Training and Research Hospital, Istanbul, Turkey; 132 University College London, London, UK; 133 University of Plymouth, Plymouth, UK; 134 Parkinson’s UK, London, UK; 135 University of Glasgow, Glasgow, UK; 136 Queen Mary University of London, London, UK; 137 Cardiff University, Cardiff, UK; 138 Royal Veterinary College, University of London, London, UK; 139 University of Bristol, Bristol, UK; 140 Cure Parkinson’s Trust, London, UK; 141 University of Cincinnati, Cincinnati, OH USA; 142 The Michael J. Fox Foundation for Parkinson’s Research, New York, NY USA; 143 Augusta University / University of Georgia Medical Partnership, Athens, GA USA; 144 Mid-Atlantic Permanente Medical Group, Rockville, MD USA; 145 Washington University in St. Louis, St. Louis, MO USA; 146 National Institutes of Health, Bethesda, MD USA; 147 Indiana University, Bloomington, IN USA; 148 Rush University Medical Center, Chicago, IL USA; 149 Kaiser Permanente, Oakland, CA USA; 150 Aligning Science Across Parkinson’s, Washington, DC USA; 151 Banner Sun Health Research Institute, Sun City, AZ USA; 152 Michigan State University, East Lansing, MI USA; 153 Northwestern University, Chicago, IL USA; 154 Baylor College of Medicine, Houston, TX USA; 155 Baylor College of Medicine/Texas Children’s Hospital, Houston, TX USA; 156 University of Miami Miller School of Medicine, Miami, FL USA; 157 Beth Israel Deaconess Medical Center, Boston, MA USA; 158 NorthShore University HealthSystem, Evanston, IL USA; 159 Institute for Neurodegenerative Disorders, New Haven, CT USA; 160 University of Pittsburgh, Pittsburgh, PA USA; 161 University of Alabama at Birmingham, Birmingham, AL USA; 162 University of Maryland School of Medicine, Baltimore, MD USA; 163 University of Michigan, Ann Arbor, MI USA; 164 Columbia University Irving Medical Center, New York, NY USA; 165 James J. Peters VA Medical Center, Bronx, NY USA; 166 University of Chicago, Chicago, IL USA; 167 Indiana University School of Medicine, Indianapolis, IN USA; 168 Hue University, Huế, Vietnam; 169 University of Zambia, Lusaka, Zambia

**Keywords:** Genomics, Structural variation, Parkinson's disease

## Abstract

Risk prediction models play a crucial role in advancing healthcare by enabling early detection and supporting personalized medicine. Nonetheless, polygenic risk scores (PRS) for Parkinson’s disease (PD) have not been extensively studied across diverse populations, contributing to health disparities. In this study, we constructed 105 PRS using individual-level data from seven ancestries and compared two different models. *Model 1* was based on the cumulative effect of 90 known European PD risk variants, weighted by summary statistics from four independent ancestries (European, East Asian, Latino/Admixed American, and African/Admixed). *Model 2* leveraged multi-ancestry summary statistics using a *p*-value thresholding approach to improve prediction across diverse populations. Our findings provide a comprehensive assessment of PRS performance across ancestries and highlight the limitations of a “one-size-fits-all” approach to genetic risk prediction. We observed variability in predictive performance between models, underscoring the need for larger sample sizes and ancestry-specific approaches to enhance accuracy. These results establish a foundation for future research aimed at improving generalizability in genetic risk prediction for PD.

## Introduction

The heritability attributed to idiopathic Parkinson’s disease (PD) in European populations is estimated to be around 22%^1^. Genome-wide association studies (GWAS) have been key in identifying common loci that contribute to PD risk. A total of 90 risk variants across 78 independent loci have been associated with PD risk in European ancestry populations^1^. More recently, large-scale efforts are focusing on increasing genetic diversity in PD to unravel the genetic architecture of the disease across ancestries^2–5^. The first and largest multi-ancestry PD GWAS meta-analysis performed to date in European, East Asian, Latino/Admixed American, and African ancestry populations identified a total of 78 loci reaching genome-wide significance, 12 of which had not been previously identified^6^.

A polygenic risk score (PRS) can be generated to estimate an individual’s susceptibility to a binary or a continuous outcome, exploring the cumulative estimated effect of common genetic variants on an individual’s phenotype, like PD^7,8^. In this context, PRS alone has not been shown to have clinical utility in predicting PD risk in European populations, with only 56.9% sensitivity and 63.2% specificity to predict disease at best^9^. PRS utility improves both sensitivity (83.4%) and specificity (90.3%) when including relevant clinical data elements such as olfactory function, family history, age, and gender^9,10^. Similarly, the integration of environmental factors, multi-omics data, and clinical criteria in PRS models boosts performance across multiple diseases^11–13^.

Nevertheless, the current focus on European ancestries in PRS development highlights a significant research gap. While recent studies^2,14–16^ have begun to explore the application of PRS in PD across variable genetic ancestries, the predominant reliance on European datasets may introduce limits on model generalizability. Using PRS to calculate disease risk in a single population may exacerbate the performance of the model(s) when applied globally across ancestries^17,18^.

**Fig. 1:**
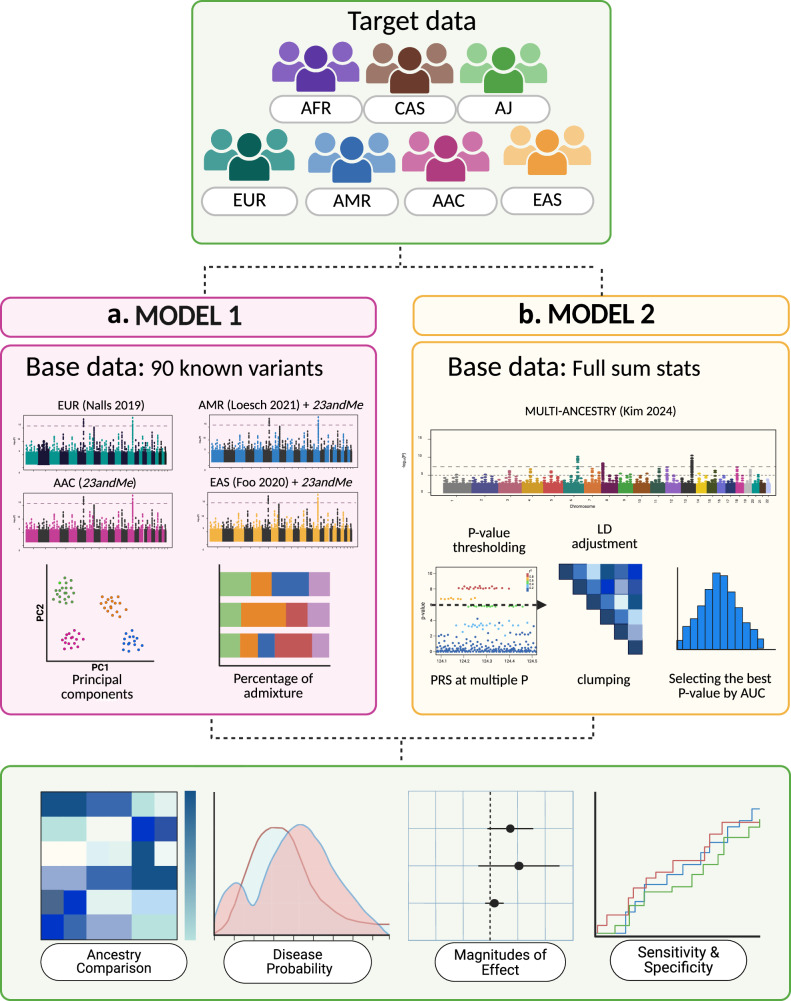
Schematic study workflow. The study workflow is summarized in three panels. The first panel presents the individual-level datasets (*target data*) from seven diverse ancestry groups: African Admixed (AAC), African (AFR), Ashkenazi Jewish (AJ), Latino/Admixed American (AMR), Central Asian (CAS), East Asian (EAS), and European (EUR). The second panel compares the two implemented models: **a** *Model 1* evaluates the cumulative effect of the 90 Parkinson’s disease (PD) risk variants identified by Nalls et al.^1^, across the *target data*, weighted by effect sizes from four population-specific summary statistics (*base data*) (EUR, AAC, AMR, EAS) and adjusted by principal components or percentage of ancestral admixture, leading to the generation of 56 scores; and **b** *Model 2* implements a best-fit *p*-value thresholding PRS along with variant-specific weights based on the multi-ancestry summary statistics from Kim et al.^6^ (pruned using default parameters). This approach generated a total of 49 PRS. The third panel includes visualizations used to interpret results: heatmaps for ancestry comparison, density plots for disease probability, forest plots for effect size, and Receiver Operating Characteristic (ROC) plots to evaluate model sensitivity and specificity.

Here, we conduct a broad assessment of PRS in PD, comparing seven ancestries and applying two different methodological approaches, which are summarized and visualized in Fig. 1. The first approach (here referred to as *Model 1)* examines the cumulative effect of the 90 known European PD risk variants, leveraging population-specific (European, East Asian, Latino/Admixed American, and African Admixed populations) effect sizes derived from four summary statistics (*base data*) (Supplementary Table 1a). This approach implements PRS models on non-overlapping individual-level data from the Global Parkinson’s Genetics Program (GP2) (*target data*) across seven ancestries (East Asian, Central Asian, Latino/Admixed American, African, African Admixed, European and Ashkenazi Jewish) (Table 1, Supplementary Fig. 1). As part of *Model 1*, we further investigated potential differences in model performance when adjusting for principal components or the percentage of ancestral admixture. The second approach (here referred to as *Model 2)* utilized summary statistics from a recent PD multi-ancestry GWAS meta-analysis (Supplementary Table 1b) while applying a best-fit *p*-value thresholding approach to the same individual-level ancestry (*target data*)^6^. By doing so, we aimed to explore risk variability across a global survey of genetic ancestries and evaluate the accuracy and effectiveness of these models. Due to insufficient clinical data on olfactory function, family history, and age across diverse ancestries, we did not include these predictors in our models.

**Table 1:** Demographic and clinical characteristics of the studied cohorts

			Cases		Controls	
Cohort	Total	Male (*n*, %)	*n*	AAO (mean ± SD)	*n*	Age (mean ± SD)
EUR	31799	18936 (59.54%)	22093	58.94 ± 11.75	9706	62.39 ± 13.01
AAC	1144	476 (41.60%)	325	58.60 ± 12.29	819	64.89 ± 11.45
AMR	3358	1590 (47.35%)	1928	54.34 ± 13.54	1430	59.91 ± 8.47
EAS	4167	2683 (64.39%)	1819	56.85 ± 12.35	2348	62.42 ± 11.09
AFR	2606	1440 (55.25%)	954	57.08 ± 12.73	1652	63.08 ± 15.46
AJ	1819	1228 (67.50%)	1396	62.46 ± 11.87	423	67.78 ± 9.80
CAS	905	408 (45.08%)	582	53.43 ± 11.46	323	54.98 ± 6.11

For controls, age represents age at sample collection, and for cases, age at onset (AAO), both presented as mean ± Standard Deviation (SD).

*AFR* African, *AJ* Ashkenazi Jewish, *CAS* Central Asian, *AAC* African Admixed, *AMR* Latino/Admixed American, *EAS* East Asian, *EUR* European.

## Results

### Risk estimates show expected high levels of heterogeneity in predicting disease status across diverse ancestry populations

**Fig. 2:**
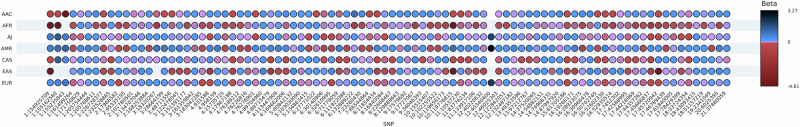
Upset plot showing risk heterogeneity across ancestries. Case-control association analysis results for the 90 risk variants across ancestries. The Y-axis lists the ancestry populations — African Admixed (AAC), African (AFR), Ashkenazi Jewish (AJ), Latino/Admixed American (AMR), Central Asian (CAS), East Asian (EAS), and European (EUR)—while the X-axis shows the 90 risk variants. The color bar indicates the magnitude of effect as the log of the odds ratio (beta value) and its directionality, with red representing negative directionality and blue representing positive directionality, after standardizing the effect allele for each estimate. Note: the directionality of effect for variants with non-significant association *p*-values (>0.05) should be interpreted with caution and considered only as a potential trend. Variant *p*-values can be found in Supplementary Table 3. Empty slots represent variants that were not present in cases or controls within the corresponding ancestry.

In analyzing the distribution patterns of the 90 lead SNPs contributing to risk from Nalls et al.^1^ across the seven ancestry cohorts under study, we observed significant heterogeneity among these predictors. Differences between ancestries included the number of valid predictors (defined as the subset of the 90 independent variants present in both the base and *target data* across ancestries) (Supplementary Table 2), directionality, variant frequency, and magnitude of effect, suggesting substantial population-specific divergences in the genetic architecture of disease (Fig. 2). The magnitudes of effect and *p*-values, which offer context regarding the significance and directionality of each variant’s effect, are quantified in Supplementary Table 3. Of note, the number of valid predictors for PRS was found to be fewer than 90 in many non-European populations. Variants contributing to PRS in European populations may be rare in other ancestries, making them difficult to impute accurately. Furthermore, these variants may not align with haplotypes associated with PD risk across different ancestries due to variations in linkage disequilibrium (LD) patterns, highlighting the existence of diverse genetic architectures for disease risk.

### *Model 1* performance across diverse ancestries

**Fig. 3:**
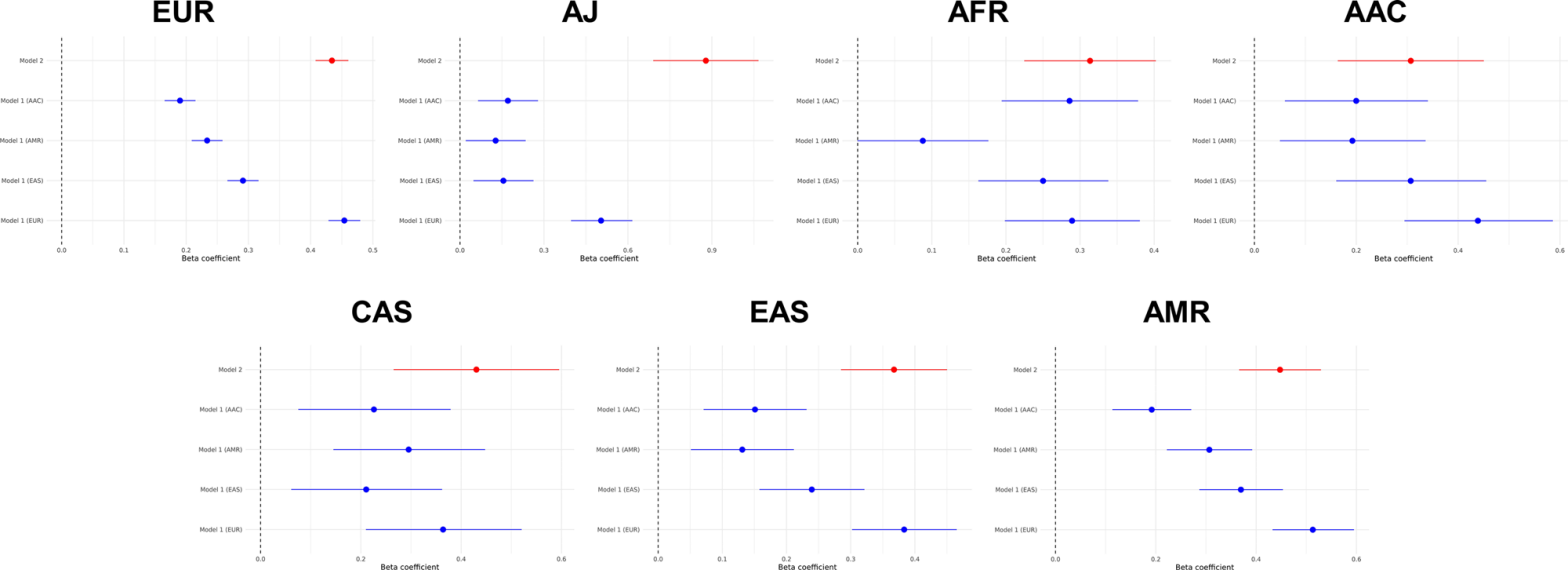
*Model 1* and *Model 2* magnitude of effect for each cohort. Forest plots comparing the effectiveness of risk prediction across the studied ancestries. Each panel contrasts individual-level data for the cohorts under study with the *Model 1* population-specific summary statistics — European (EUR), East Asian (EAS), Latino/Admixed American (AMR), and African Admixed (AAC) — as well as the multi-ancestry data used in *Model 2*. The X-axis represents the magnitude of effect, while the Y-axis lists the summary statistics for each group. The dots symbolize the value of the beta coefficient, and the horizontal lines depict the 95% confidence intervals.

European GWAS-derived PRS models utilizing the 90 risk predictors and their effect estimates from Nalls et al.^1^, and adjusted by sex, age, and 10 principal components (PCs), exhibited variable predictive accuracy across ancestries (Table 2, Fig. 3, Supplementary Figs. 2 and 3). Interestingly, these models generally outperformed PRS models that leveraged summary statistics from non-European populations, even when implemented on the same population-specific predicted ancestry cohorts. This observation further reinforces our hypothesis, as (1) the 90 lead SNPs that contribute to PD in Europeans do not capture the complexity underlying risk haplotypes in non-European populations, and (2) non-European summary statistics are underpowered to detect meaningful effects. In the European population (positive control), this model achieved an area under the curve (AUC) of 0.63 with a balanced accuracy of 0.59 (Table 2), confirming the expected predictability in this cohort^1^. The Ashkenazi Jewish population exhibited the highest AUC of 0.66 with a balanced accuracy of 0.62 (Table 2), reflecting strong predictive capability.

**Table 2:** *Model 1* performance across ancestries

Target data	Base data	AUC	Accuracy (95% CI)	Balanced Accuracy	Sensitivity	Specificity
**EUR**	AAC	0.554	0.531 (0.525–0.536)	0.539	0.517	0.562
	AMR	0.569	0.556 (0.551–0.562)	0.553	0.561	0.545
	EAS	0.584	0.558 (0.552–0.563)	0.561	0.553	0.570
	EUR	0.632	0.596 (0.590–0.601)	0.595	0.597	0.594
**AAC**	AAC	0.585	0.565 (0.535–0.594)	0.571	0.585	0.557
	AMR	0.575	0.612 (0.583–0.640)	0.568	0.468	0.669
	EAS	0.622	0.578 (0.549–0.607)	0.604	0.665	0.543
	EUR	0.651	0.617 (0.588–0.645)	0.612	0.600	0.624
**AMR**	AAC	0.579	0.561 (0.544–0.578)	0.563	0.553	0.573
	AMR	0.505	0.507 (0.490–0.524)	0.511	0.483	0.538
	EAS	0.625	0.594 (0.577–0.611)	0.592	0.607	0.577
	EUR	0.636	0.597 (0.580–0.614)	0.594	0.613	0.576
**EAS**	AAC	0.58	0.558 (0.542–0.573)	0.560	0.577	0.542
	AMR	0.537	0.538 (0.523–0.554)	0.531	0.472	0.590
	EAS	0.55	0.539 (0.523–0.554)	0.538	0.529	0.546
	EUR	0.618	0.586 (0.571–0.601)	0.590	0.621	0.558
**AFR**	AAC	0.543	0.540 (0.521–0.560)	0.537	0.526	0.548
	AMR	0.511	0.520 (0.501–0.539)	0.506	0.455	0.558
	EAS	0.536	0.559 (0.540–0.579)	0.535	0.445	0.625
	EUR	0.536	0.507 (0.488–0.526)	0.522	0.579	0.465
**AJ**	AAC	0.543	0.504 (0.480–0.527)	0.535	0.476	0.593
	AMR	0.545	0.506 (0.483–0.530)	0.553	0.466	0.641
	EAS	0.556	0.557 (0.534–0.580)	0.548	0.564	0.532
	EUR	0.665	0.615 (0.592–0.637)	0.624	0.607	0.641
**CAS**	AAC	0.557	0.562 (0.529–0.595)	0.553	0.586	0.520
	AMR	0.585	0.556 (0.523–0.588)	0.565	0.533	0.598
	EAS	0.566	0.536 (0.503–0.569)	0.548	0.505	0.591
	EUR	0.591	0.591 (0.558–0.623)	0.579	0.622	0.536

Accuracy metrics for each instance of *Model 1*, including area under the curve (AUC), accuracy with 95% confidence interval (95% CI), balanced accuracy, sensitivity, and specificity for each target dataset, along with corresponding population-specific *base data*: *AFR* African, *AJ* Ashkenazi Jewish, *CAS* Central Asian, *AAC* African Admixed, *AMR* Latino/Admixed American, *EAS* East Asian, *EUR* European.

Despite having the lowest number of valid predictors (87) among the studied population-specific cohorts, European GWAS-derived *Model 1* implemented on the East Asian population cohort achieved an AUC of 0.62, odds ratio (OR) of 1.47 (95% CI: 1.35–1.59), and balanced accuracy of 0.59 (Table 2, Supplementary Tables 2 and 4). This implies that a well-chosen set of predictors can be more impactful than simply increasing the number of variables included in the model. In contrast, the performance of *Model 1* in African ancestries was the lowest, with an AUC of 0.54, OR of 1.34 (95% CI: 1.22–1.46), and balanced accuracy of 0.54 (Table 2, and Supplementary Table 4). Of note, the European-centric model performed particularly well on African Admixed individuals, exhibiting an AUC of 0.65, OR of 1.55 (95% CI: 1.34–1.80), and balanced accuracy of 0.61 (Table 2, and Supplementary Table 4). This may be due in part to their relatively high percentage of European admixture, thus showing stronger alignment with European genetic markers. This observation would further support the hypothesis that the performance of these models can be influenced by how closely the individuals in the sample resemble the reference population on which the model was trained.

Further analyses were conducted to determine the individual effect size contributions of genetic variants to *Model 1* using summary statistics from four ancestries independently within each population. These analyses, detailed in Supplementary Table 5 for the top five hits per model, uncovered differences among the 90 variants not only in effect size, as depicted in Fig. 2 and Supplementary Table 3, but also in the extent to which they influence PRS for each population. Of note, *SNCA* (rs356182) emerges among the strongest predictors across ancestries. The effect of *SNCA* was most prominent for PRS models when using European and African Admixed summary statistics, as well as with Latino/Admixed American summary statistics, but was notably absent altogether from the meta-analyzed East Asian summary statistics. LD differences could account for this observation in the East Asian population, consistent with Foo et al.^2^, who nominated *SNCA* rs6826785 as the top GWAS hit underlying this locus association. This signal has an *R*^2^ = 0.479 with *SNCA* rs356182, indicating a moderate correlation. Indeed, applying *Model 1* on the East Asian cohort using only the 11 risk variants identified by Foo et al.^2^ produced a balanced accuracy of 0.573, AUC of 0.60, and OR of 1.35 (95% CI: 1.25–1.46), thus performing better than the original model using the 87 predictors from Nalls et al.^1^ (Table 2, Supplementary Table 4).

As expected, *LRRK2* G2019S (rs34637584) was found to be among the most relevant predictors in the European, Ashkenazi Jewish, and Latino/Admixed American populations only when European summary statistics were incorporated into the model, as it was absent from each other ancestry-specific summary statistics. This is most likely explained by statistical power differences, given that *LRRK2* G2019S is a less frequent variant compared to more common GWAS hits, and the European summary statistics were the most well-powered. As anticipated, *GBA1* N370S (rs76763715) and *GBA1* E326K (tagged by rs35749011) were found to make significant contributions in the Ashkenazi Jewish and European populations, respectively. The strong predictive power of the European *base data* for the Ashkenazi Jewish population is likely explained by the higher frequency of *LRRK2* G2019S and *GBA1* N370S carriers within this population (Supplementary Table 5).

We aimed to further adjust for the potential variability driven by ancestral admixture patterns. The results, displayed in Supplementary Fig. 4 and Supplementary Table 4, show that the model adjusted by PCs and the model adjusted by admixture remain consistent across ancestries. This suggests that adjusting for the percentage of admixture does not provide additional benefits over PCs for the populations assessed.

### *Model 2* performance across diverse ancestries

**Fig. 4:**
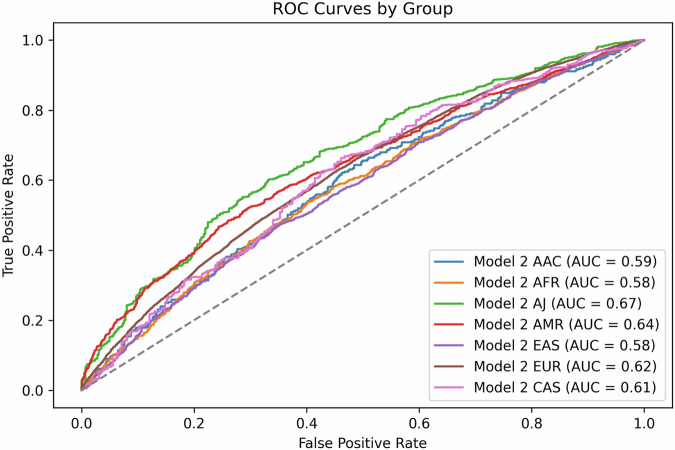
*Model 2* performance for each cohort. Receiver operating characteristic (ROC) curves evaluating the performance of *Model 2*. Each cohort is represented by a color-coded curve: African Admixed (AAC) in blue, African (AFR) in orange, Ashkenazi Jewish (AJ) in green, Latino/Admixed American (AMR) in red, East Asian (EAS) in purple, European (EUR) in brown, and Central Asian (CAS) in pink. The Y-axis represents the true positive rate (sensitivity), and the X-axis shows the false positive rate (1-specificity).

*Model 2*, based on a best-fit *p*-value thresholding approach using summary statistics from the multi-ancestry GWAS meta-analysis conducted by Kim et al.^6^ (see “Methods” section for a detailed explanation), demonstrates varied effectiveness across ancestries (Table 3, Figs. 3 and 4, Supplementary Figs. 5 and 6). Several trends stand out from the optimal *p*-value thresholds identified here. First, the number of valid independent predictors selected by PRSice for *Model 2* is higher in populations with historically smaller haplotype blocks compared to other populations at the same *p*-value thresholds. For example, African populations tend to have smaller haplotypes than European populations due to their greater genetic diversity, higher recombination rates, and longer evolutionary history. In contrast, European populations have longer haplotype blocks, resulting from genetic bottlenecks and lower historical recombination rates. This is reflected by the elevated number of independent SNPs included at a threshold of *p* = 5e-07 for the African population (506) than for the European population (267) (Table 3).

**Table 3:** *Model 2* performance across ancestries

Cohort	Threshold	PRS *R*^2^ adj	Full *R*^2^	Null *R*^2^	Coefficient	SE	No. of SNP	OR (95% CI)	AUC	Accuracy (95% CI)	Balanced accuracy	Sensitivity	Specificity
AAC	5.00E-08	0.012	0.122	0.112	0.307	0.073	273	1.36 (1.18–1.57)	0.591	0.561 (0.532–0.59)	0.579	0.622	0.537
AFR	5.00E-07	0.012	0.097	0.086	0.314	0.045	506	1.37 (1.25–1.50)	0.583	0.576 (0.557–0.595)	0.568	0.538	0.599
AJ	5.00E-06	0.038	0.063	0.026	0.879	0.096	457	2.41 (1.99–2.91)	0.67	0.617 (0.595–0.64)	0.635	0.602	0.667
AMR	5.00E-08	0.025	0.193	0.172	0.448	0.042	206	1.56 (1.44–1.70)	0.639	0.605 (0.588–0.621)	0.608	0.586	0.629
EAS	5.00E-08	0.015	0.256	0.244	0.368	0.042	223	1.44 (1.33–1.57)	0.578	0.556 (0.54–0.571)	0.556	0.557	0.555
EUR	5.00E-07	0.021	0.048	0.027	0.434	0.013	267	1.54 (1.50–1.59)	0.621	0.598 (0.592–0.603)	0.586	0.615	0.557
CAS	5.00E-06	0.018	0.091	0.075	0.43	0.084	485	1.54 (1.30–1.81)	0.612	0.604 (0.572–0.636)	0.598	0.62	0.576

Metrics shown here include the best *p*-value threshold for SNP inclusion (Threshold), the variance in the target phenotype explained by the PRS adjusted by a prevalence set to 0.005 (PRS *R*² adj), the variance explained by the full-model regression that includes covariates (Full *R*²), and the variance explained by the covariates alone (Null *R*²). Also provided are the regression coefficient (Coefficient), Standard Error (SE), the number of SNPs included in the PRS (No. of SNP), the odds ratios with 95% confidence intervals (OR [95% CI]), and the area under the curve (AUC).

*AFR* African, *AJ* Ashkenazi Jewish, *CAS* Central Asian, *AAC* African Admixed, *AMR* Latino/Admixed American, *EAS* East Asian, *EUR* European.

Additionally, more stringent *p*-value thresholds appear to produce the best-performing models in populations with generally smaller haplotype blocks, such as admixed populations. At more lenient thresholds, admixed populations like African Admixed and Latino/Admixed Americans exhibited overfitting (Supplementary Fig. 6). This may be due in part to the representation of these ancestries in the *base data* – each of the ancestries for which the most stringent (5e-08) *p*-value threshold yielded the best-fit model (East Asian, African Admixed, and Latino/Admixed American) were included in the multi-ancestry summary statistics from Kim et al.^6^ It is, thus, more likely that risk variants contributing to disease in these populations would be found to be significant in the *base data,* while prediction models for other ancestries would require the inclusion of more SNPs.

**Fig. 5:**
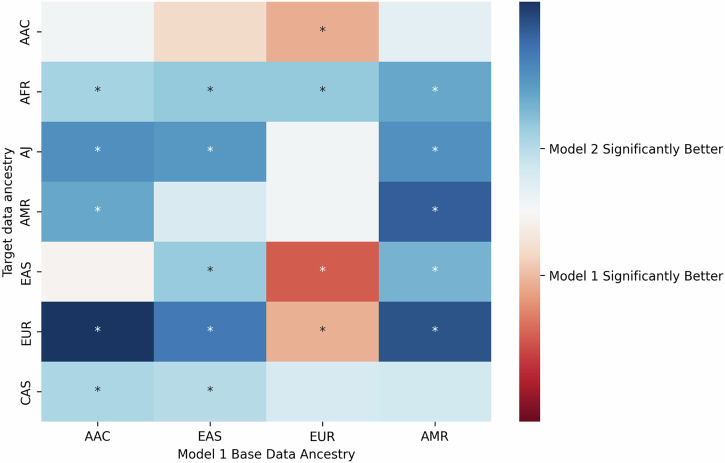
Comparison of polygenic risk score performance between *Model 1* and *Model 2.* Heatmap comparing the performance of the two models under study based on DeLong’s test. The X-axis represents the *base data* for *Model 1* being compared against *Model 2*, with ancestry-specific summary statistics adjusted by principal components (PCs), while the Y-axis indicates the *target data*. The seven ancestry groups analyzed include African (AFR), Ashkenazi Jewish (AJ), Central Asian (CAS), African Admixed (AAC), Latino/Admixed American (AMR), East Asian (EAS), and European (EUR). The color scale represents the difference in AUC performance between the two models, ranging from red (*Model 1* performs better) to blue (*Model 2* performs better). Asterisks (*) indicate statistically significant differences (*p* < 0.05) in performance between the models.

Overall, *Model 2* performed best on the Ashkenazi Jewish cohort and worst on the African and East Asian cohorts, with AUCs ranging from 0.58 to 0.67 (Table 3, Fig. 4). These results are comparable to the European *base data* implementation of *Model 1*, with *Model 1* generally performing better on East Asian, African Admixed, and European populations, *Model 2* performing better on Ashkenazi Jewish, Central Asian, and African populations, and inconclusive results with the Latino/Admixed American cohort based on ORs and balanced accuracy (Tables 2 and 3, Supplementary Table 4). When comparing AUCs using DeLong’s test, *Model 1* performs significantly better for three ancestries (European, East Asian, and African Admixed) while *Model 2* performs significantly better for the African ancestry (Fig. 5, Supplementary Table 6).

Alternatively, *Model 2* consistently outperforms *Model 1* when using non-European *base data*. Notably, *Model 2* produced more robust predictions than *Model 1* weighted by East Asian, African, and Latino/Admixed American *base data* when applied to those three ancestries, respectively, based on ORs, AUCs, and balanced accuracies (Tables 2 and 3, Fig. 5, Supplementary Fig. 5, and Supplementary Table 4). This parallels aforementioned findings from *Model 1* using European *base data*, similarly underscoring the importance of utilizing well-powered summary statistics in PRS analyses.

## Discussion

This study represents the first comprehensive assessment of PRS in predicting PD risk across diverse ancestries. While previous genetic research has primarily focused on populations of European ancestry, our study builds upon this by providing an extensive global landscape of PRS contributing to PD. We employed two distinct methodological approaches for PRS calculation: *Model 1* focused on 90 European-centric risk variants while leveraging four population-specific summary statistics, and *Model 2* was based on best-fit *p*-value thresholding applied to multi-ancestry summary statistics. Additionally, we tested various covariate adjustments (principal components *versus* percentage of admixture) and utilized different base datasets (single population-specific summary statistics *versus* combined multi-ancestry GWAS meta-analysis).

Our study revealed that while our understanding of PD risk is predominantly derived from European genetic studies, *Model 1,* utilizing summary statistics from Europeans, shows to some extent applicability across diverse populations, including Ashkenazi Jewish (harboring certain levels of European ancestry and enriched with *LRRK2* and *GBA1* carriers) and East Asians. Of note, PRS models derived from the 90 risk predictors originating from European populations and constructed using estimates from population-specific summary statistics failed to enhance predictability. We hypothesized that population-specific summary statistics for a given ancestry would not necessarily outperform European-based PRS models when estimating the cumulative effect of the 90 risk variants. Our findings support this hypothesis, reinforcing the notion that unique population-specific haplotypes contribute to PD risk across populations. This underscores the importance of addressing the current scarcity of robust population-specific summary statistics.

We sought to reconcile these discrepancies and enhance our ability to forecast risk by devising a best-fit multi-ancestry PRS approach based on *p*-value thresholding, leveraging multi-ancestry GWAS data to select the best set of cumulative SNPs. This approach yields similar results to *Model 1* European-specific summary statistics (performing better on the African cohort and worse on the European, East Asian, and African Admixed cohorts), while performing significantly better than models using non-European summary statistics across nearly all cohorts. This performance exemplifies the challenge that a ‘one size fits all’ approach presents in genetic research, advocating for more nuanced strategies in precision medicine that account for more global genetic variability. While both population specificity and statistical power of base datasets seem to contribute to predictive accuracy, the comparatively strong performance of *Model 2* against ancestry-specific implementations of *Model 1* suggests the latter may be the prevailing factor.

The results observed in East Asians align with the work reported by Foo et al.^2^ and support the cross-population applicability of PRS in PD, which has already been evidenced in this population in the context of Alzheimer’s disease^19^, breast cancer^20^, and colorectal cancer^21^. The major contributor for the PRS in this cohort was *SNCA* (rs356182), with an absolute mean effect twice as high as *LRRK2* G2019S, the most significant SNP in Europeans (Supplementary Table 5). Independent analysis of the 11 risk variants identified by Foo et al.^2^ is consistent with their reported findings and suggests improved performance when using ancestry-specific datasets. Specifically, this is likely due in large part to the inclusion of *SNCA* rs6826785, which was not present in the summary statistics from Nalls et al.^1^ but was shown to be a significant risk factor within East Asian populations^2^. This result is particularly compelling as European and East Asian genetic ancestries are very different, as illustrated in ancestry prediction models (Supplementary Fig. 1), contrasting with the hypothesis that the accuracy of PRS depends on genetic ancestry proximity^17^.

Several limitations should be acknowledged. First, the summary statistics here are substantially comprised of *23andMe* self-reported cases and UK Biobank proxy cases. Although Nalls et al.^1^ reported strong genetic correlations between summary statistics that include PD cases ascertained by clinicians compared to *23andMe* self-reported cases (genetic correlation from LDSC (rG) = 0.85, SE = 0.06) and UKB proxy cases (rG = 0.84, SE = 0.134), the inclusion of non-clinically diagnosed cases may be diluting PRS accuracy to predict disease across all ancestries. Another limitation, given the scarcity of heritability estimates, disease prevalence, and summary statistics from non-European data, is that our power calculations were derived based on estimates from European populations. Consequently, these estimates may lead to biases in the sample size required to predict disease status across diverse ancestries. Another important constraint is the absence of individual-level replication datasets per ancestry. The lack of replication data hampers the robustness and generalizability of our findings across different individual-level datasets from diverse ancestral populations. Additionally, we acknowledge that different ancestry prediction approaches were used for the *23andMe* datasets that were meta-analyzed here, which may have introduced intra-ancestry heterogeneity. A significant limitation in conducting PRS for highly admixed populations, such as Latino/Admixed Americans, is the genetic diversity across regions, including Caribbean Hispanics, Central Americans, and South Americans. The lack of subpopulation reference panels prevents the separate assessment of these distinct genetic clusters, reflecting the current constraints in available data. Finally, although the meta-analyzed *base data* used for *Model 2* featured multiple diverse ancestries, 83% of the PD cases are of European ancestry.

To address these limitations, future research should prioritize larger sample sizes for individual-level datasets per ancestry and subpopulation within ancestries, as well as the availability of well-powered ancestry-specific summary statistics. Incorporating local ancestry estimates into PRS^22^ could substantially improve performance in highly admixed populations. This approach allows for the use of summary statistics from the ancestry PRS panel corresponding to the specific chromosomal region of the individual under risk inference, mitigating inflation or deflation caused by ancestry-specific risk alleles. Additionally, methods like PRS-CSx^23^ can integrate data from multiple sets of summary statistics across different ancestries^24^. These offer a promising avenue for improving the transferability and accuracy of PRS models in diverse populations.

Studying biomarker-defined PD cohorts, rather than those diagnosed solely by clinical criteria, is also crucial. At least 5% of individuals diagnosed with PD do not demonstrate neuronal alpha-synuclein, a hallmark required for definitive diagnosis^25^. Employing multi-modality machine learning (ML) approaches^11^ that combine adjusted transcriptomics, genetics, and clinical data into a predictive model could provide a more comprehensive understanding of PD risk and improve prediction accuracy globally. By leveraging complex patterns not evident in isolated data modalities, ML algorithms such as deep learning may improve risk prediction, ultimately enabling more personalized strategies for prevention, diagnosis, and treatment.

This study presents a comprehensive evaluation of 105 PRS models for PD risk across seven diverse ancestries, including admixed and underrepresented populations. Our analysis highlights the heterogeneity of PD risk factors and underscores the bias introduced by predominantly European-derived genetic data. While some European-based PRS models demonstrated transferability to other ancestries, their performance varied significantly across populations, emphasizing the need for larger and more diverse datasets. Acknowledging these limitations, our results provide data-driven evidence of the diverse genetic architecture of PD and lay the groundwork for future research.

## Methods

Our study workflow is highlighted in Fig. 1. We obtained multi-ancestry individual-level data from the Global Parkinson’s Genetics Program (GP2)^5^ release 9 (10.5281/zenodo.7904831). These data (here referred to as *target data*) were used to test PRS models and comprised a total of 50,234 participants, including 31,985 individuals diagnosed with PD according to the Movement Disorder Society (MDS)^26^ or Queen Square Brain Bank (QSBB) diagnostic criteria^27^, and 18,249 controls. After excluding locally-restricted samples and related individuals (those at the first cousin level or closer) that could bias our PRS assessments, our dataset comprised a total of 45,799 individuals, of which 29,097 were PD cases and 16,702 controls. The following genetic ancestries were included: African Admixed, African, Ashkenazi Jewish, Latino/Admixed American, Central Asian, East Asian, and European populations (Supplementary Fig. 1). Detailed demographic and clinical characteristics can be found in Table 1.

We performed genotype data generation according to standard protocols from GP2^5^ release 9. In summary, samples were genotyped on the NeuroBooster array^28^ (v.1.0, Illumina, San Diego, CA) that includes 1,914,935 variants encompassing ancestry informative markers, markers for identity-by-descent determination, and X-chromosome SNPs for sex determination. Additionally, the array includes 96,517 customized variants. Automated genotype data processing was conducted on GenoTools^29^, a Python pipeline built for quality control (QC) and ancestry estimation of data. Additional details can be found at https://pypi.org/project/the-real-genotools/^29^. There was no overlap between the base and *target data* used in our study. We ensured that all *base data* used for PRS calculation were entirely independent of the population-specific individual-level data.

QC was conducted following standard protocols, with adjustments made to enhance precision and reliability. Samples exhibiting a genotype call rate below 98% (--mind 0.02), discordant sex determinations (0.25 ≤ sex *F* ≤ 0.75), or significant heterozygosity (*F* ≤ −0.25 or *F* ≥ 0.25) were excluded from the analysis. Additional QC measures involved the exclusion of SNPs with a missingness rate above 2%, variants deviating significantly from Hardy-Weinberg Equilibrium (HWE *P*-value < 1E-4), and variants showing non-random missingness by case-control status (*P* ≤ 1E-4) or by haplotype (*P* ≤ 1E-4 per ancestry).

Ancestry predictions were refined using an updated and expanded reference panel, which, as of February 2025, comprises samples from the 1000 Genomes Project (https://www.internationalgenome.org/data-portal/data-collection/phase-1)^30^, Human Genome Diversity Project^31^, and an Ashkenazi Jewish population dataset^32^. This panel includes 819 African, 74 African Admixed and Caribbean, 471 Ashkenazi Jewish, 183 Central Asian, 585 East Asian, 534 European, 99 Finnish, 490 Latino/Admixed American, 152 Middle Eastern, and 601 South Asian individuals. Palindromic SNPs were excluded to improve accuracy (AT/TA or GC/CG). The process ensured that the variants for ancestry estimations, overlapping between the reference SNP set panel and the genotyping data from the samples under study, were subjected to the same QC criteria as all other remaining variants, including exclusion of palindromic SNPs, filtering for MAF below 0.05, genotyping call rate less than 0.98, and HWE *p*-value less than 1E-4. Missing genotypes were imputed using the mean value of the variant from the reference panel.

To evaluate the efficacy of ancestry estimation, an 80/20 train/test split was applied to the reference panel samples, and PCs were calculated using the overlapping SNPs. By applying transformations through UMAP, the global genetic population substructure and stochastic variation were visualized. Training a linear support vector classifier on the UMAP-transformed PCs resulted in consistent predictions, with balanced accuracies between 95% and 98%, as verified by 5-fold cross-validation on the test data from the reference panel. These classifier models were then applied to the dataset to generate ancestry estimates for all samples. Detailed methodologies for the cloud-based and scalable pipeline employed for genotype calling, QC, and ancestry estimation are documented in the GenoTools^29^ GitHub repository (10.5281/zenodo.10719034).

Following ancestry estimation, we excluded those with second-degree or closer relatedness (kinship coefficient > 0.0884). PCs that were used as covariates in the PRS analysis were recalculated per ancestry post-QC and ancestry determination. The percentage of ancestry was then computed using the supervised functionality of Neural ADMIXTURE (https://github.com/ai-sandbox/neural-admixture), leveraging the labeled reference panel data to estimate ancestry proportions accurately.

Variants with a MAF of less than 0.05 and HWE *p*-value less than 1E-5 were excluded before submission to the TOPMed Imputation server. The utilized TOPMed reference panel version, known as r2, encompasses genetic information from 97,256 reference samples and over 300 million genetic variants across the 22 autosomes and the X-chromosome. As of October 2023, the TOPMed panel includes approximately 180,000 participants, with 29% of African, 19% of Latino/Admixed American ancestry, 8% of Asian ancestry, and 40% of European ancestry (https://topmed.nhlbi.nih.gov/). Further details about the TOPMed Study^33^, Imputation Server^34^, and Minimac Imputation^35^ can be accessed at https://imputation.biodatacatalyst.nhlbi.nih.gov. Following imputation, the resulting files underwent pruning based on an imputation Rsq value of 0.3.

### Model 1

A total of four population-specific summary statistics (*base data*) were used to compute PRS versus the seven GP2 individual-level data ancestry cohorts (*target data*) (Supplementary Table 1a). We obtained summary statistics from the largest European PD GWAS meta-analysis to date, conducted by Nalls and colleagues (2019)^1^ (https://pdgenetics.org/resources). This study included 1,456,306 individuals, of which 1,400,000 were controls, 37,688 were cases, and 18,618 were proxy cases (defined as having a first-degree relative with PD). African Admixed summary statistics were obtained from *23andMe*, which are based on 194,273 individuals, including 193,985 controls and 288 cases. *23andMe* participants, both PD cases and controls, are self-reported and provided informed consent to participate in the research online. The study was conducted under a protocol approved by the external AAHRPP-accredited IRB, Ethical & Independent (E&I) Review Services, now part of Salus IRB (https://www.versiticlinicaltrials.org/salusirb).

In order to achieve better-powered summary statistics for the East Asian population, we meta-analyzed two independent summary statistics, including the largest East Asian PD GWAS meta-analysis to date^2^ and *23andMe* summary statistics from East Asian ancestry, which yielded a total of 183,802 individuals, including 176,756 controls and 7,046 cases. In a similar way, we conducted GWAS meta-analysis to generate better-powered Latino/Admixed American summary statistics, combining the largest Latino PD GWAS meta-analysis from the LARGE-PD Consortium^3^ with *23andMe* Latino/Admixed American summary statistics. This cohort consisted of a total of 584,660 individuals, of whom 582,220 were controls and 2440 PD cases.

Briefly, the *23andMe* data generation process could be summarized in the following steps. After the genotyping of *23andMe* participants was completed, an ancestry classifier algorithm was used to determine participant ancestries based on local ancestry and reference populations. Next, phasing was performed to reconstruct haplotypes using genotyping platform-specific panels, followed by imputation of missing genotypes, expanding the variant dataset using two independent reference panels. Related individuals were then excluded using a segmental identity-by-descent estimation algorithm to ensure unrelated participants. Finally, a GWAS analysis adjusted by covariates age, sex, and PCs was conducted, followed by GWAS QC measures to flag potential issues with SNPs, ensuring data integrity. A comprehensive explanation of each step to generate *23andMe* summary statistics, including genotyping, QC, and imputation performed by *23andMe*, can be found elsewhere^6^.

For a detailed description of the methods used to generate East Asian summary statistics, refer to the study by Foo et al.^2^ Similarly, detailed information on the Latino/Admixed American summary statistics can be found in Loesch et al.^3^ The GWAS meta-analysis of each population was carried out using fixed effects based on beta and standard error values for the 90 risk variants. This meta-analysis was conducted utilizing the METAL package, which is accessible at https://genome.sph.umich.edu/wiki/METAL_Documentation.

For *Model 1*, we extracted the lead 90 SNPs (here referred to as valid predictors) previously linked to PD risk in European ancestry populations^1^ using GP2 individual-level data for each of the seven ancestries (*target data*). Scores were weighted by the effect sizes derived from the four population-specific summary statistics previously mentioned (*base data* - European, African Admixed, Latino/Admixed American, East Asian). Logistic regression analysis was employed to predict PD status adjusted either by gender, age, and 10 PCs (28 PRS models) or by gender, age, and percentage of ancestral admixture (28 PRS models) (Fig. 1). Ancestral admixture was computed using Neural ADMIXTURE, which is described in detail at https://github.com/ai-sandbox/neural-admixture. PRS was standardized using Z-score normalization for each model. After calculating the allele counts of each variant (valid predictor) between cases and controls, we calculated the mean effect of each variant by multiplying the allele count difference by the beta coefficient, or effect size, to estimate the average impact of each variant’s allele count difference on disease phenotype. Similar approaches have been conducted in previous studies, such as Foo et al.^2^. Finally, UpSet visualizations were used to display heterogeneity estimated across known loci and multiple ancestries.

### Model 2

For *Model 2*, we used the latest multi-ancestry PD GWAS summary statistics from Kim et al.^6^, which meta-analyzed the aforementioned ancestry-specific summary statistics from four populations used in *Model 1* (Supplementary Table 1b). This comprehensive analysis yielded a total of 2,525,730 individuals, of which 49,049 were PD cases, 18,618 proxy cases, and 2,458,063 controls, highlighting the substantial scope and diversity of the data integrated into this meta-analysis.

*Model 2* was computed using PRSice-2 v2.3.5^36^. We implemented a multi-step process to estimate the cumulative genetic risk attributed to a set of SNPs based on *p*-value thresholding for each GP2 ancestry-specific cohort by using multi-ancestry GWAS summary statistics by Kim et al.^6^ (Fig. 1). PRSice-2 was used to select independent genetic variants following default PRSice-2 parameters. This approach includes adhering to standardized values (250 kb clumping window size, population-specific LD estimation using GP2 release 9 individual-level data for each population, and an LD threshold of *r*² < 0.1) as previously described^37^, and using *p*-value thresholds from 5.00e-08 to 5.00e-02, incrementing by a factor of 10 at each step. Altogether, this amounts to 49 PRS models that were developed within the framework of *Model 2*.

The *p*-value thresholding approach we implemented facilitated the evaluation of PRS predictive performance at varying levels of SNP inclusion. For each model, the PRS was calculated by summing the alleles associated with PD and weighting them by the effect sizes reported by Kim et al.^6^. Next, we determined the best-fit models by considering only *p*-value thresholds which preserved fewer SNPs than the number of participants, then selecting the model which achieved the maximal pseudo (Nagelkerke’s) *R*^2^ value, for each respective ancestry. The model was standardized using a consistent disease prevalence rate of 0.5% (0.005), as reported in previous studies^1,37^, acknowledging that these estimates are based on European data and may not generalize to other populations. This approach was necessary due to the lack of standardized or comparable prevalence rates for PD in non-European populations^38^. We further adjusted the model by sex, age, and 10 ancestry-specific PCs.

### Power calculations

To determine the cutoff for selecting a minimal sample size, we based our sample size calculation on achieving 80% power with a significance level of 0.05, using the methodology proposed by Dudbridge et al.^39^ (additional details can be found at https://github.com/DudbridgeLab/avengeme/). These initial estimates considered the 90 risk variants and the heritability reported in Nalls et al.^1^, where the heritability of PD was estimated to be 22% (*h*² = 22%) at a 0.5% disease prevalence. We determined that a minimum sample size of 550 individuals was required to reach this power threshold, assuming the limitation that our estimates are based on prevalence and heritability parameters from European populations and may not be applicable to other populations. Based on these approximations, we included only cohorts with more than 500 participants.

### Model comparisons

Results for each model were visualized through density plots displaying predicted probabilities of disease among cases, forest plots for magnitude of effects comparison, and ROC plots with associated AUC assessments. Performance metrics such as accuracy, balanced accuracy, sensitivity, and specificity were computed using the top-leftmost point of each ROC to determine probability thresholds for predicted case/control stratification of each respective model.

DeLong’s test was used to quantify statistical significance when comparing ROCs. To conduct comparisons within *Model 1*, this method was applied to each of the six combinations of *base data* ancestries. To compare *Model 1* and *Model 2*, this method was applied to each of the four Model 1 *base data* ancestries, comparing each of them independently to *Model 2*. Results for comparisons between *Model 2* and each implementation of *Model 1* were then visualized on a heatmap, with directionality indicating which model performed better and magnitude representing the degree of significance.

## Supplementary information

Supplementary Figures

Supplementary Tables

## Data Availability

Data was obtained from the Global Parkinson’s Genetics Program (GP2) and is accessible through a partnership with the Accelerating Medicines Partnership in Parkinson’s Disease (AMP-PD) and can be requested via the website’s application process (https://www.amp-pd.org/). GWAS summary statistics from GP2’s release 9 are available for all datasets. The full GWAS summary statistics for the *23andMe* discovery data are available upon application (https://research.23andme.com/dataset-access/) to qualified researchers under an agreement that protects participant privacy. These datasets are available at no cost for academic use. GenoTools (version 10; https://github.com/GP2code/GenoTools)^29^ was used for genotyping, imputation, quality control, ancestry prediction, and data processing. A secured workspace on the Verily workbench platform was created to conduct genetic analyses using GP2 release 9 data and summary statistics (https://workbench.verily.com/). Additionally, all scripts used for this study can be found in the public domain on GitHub (https://github.com/GP2code/multiancestry-PRS_PRSice; 10.5281/zenodo.11110944).

## Code availability

Data was obtained from the Global Parkinson’s Genetics Program (GP2) and is accessible through a partnership with the Accelerating Medicines Partnership in Parkinson’s Disease (AMP-PD) and can be requested via the website’s application process (https://www.amp-pd.org/). GWAS summary statistics from GP2’s release 9 are available for all datasets. The full GWAS summary statistics for the *23andMe* discovery data are available upon application (https://research.23andme.com/dataset-access/) to qualified researchers under an agreement that protects participant privacy. These datasets are available at no cost for academic use. GenoTools (version 10; https://github.com/GP2code/GenoTools)^29^ was used for genotyping, imputation, quality control, ancestry prediction, and data processing. A secured workspace on the Verily workbench platform was created to conduct genetic analyses using GP2 release 9 data and summary statistics (https://workbench.verily.com/). Additionally, all scripts used for this study can be found in the public domain on GitHub (https://github.com/GP2code/multiancestry-PRS_PRSice; 10.5281/zenodo.11110944).
