# Supplementary material for: Insights into ancestral diversity in Parkinson’s disease risk: a comparative assessment of polygenic risk scores
Source: NPJ Parkinsons Dis. 2025 Jul 3;11:201. doi: 10.1038/s41531-025-00967-4 (PMC12229533; doi:10.1038/s41531-025-00967-4)
Supplement: Supplementary file 1 — Supplementary Figures [file 41531_2025_967_MOESM1_ESM.pptx]

## Slide 1
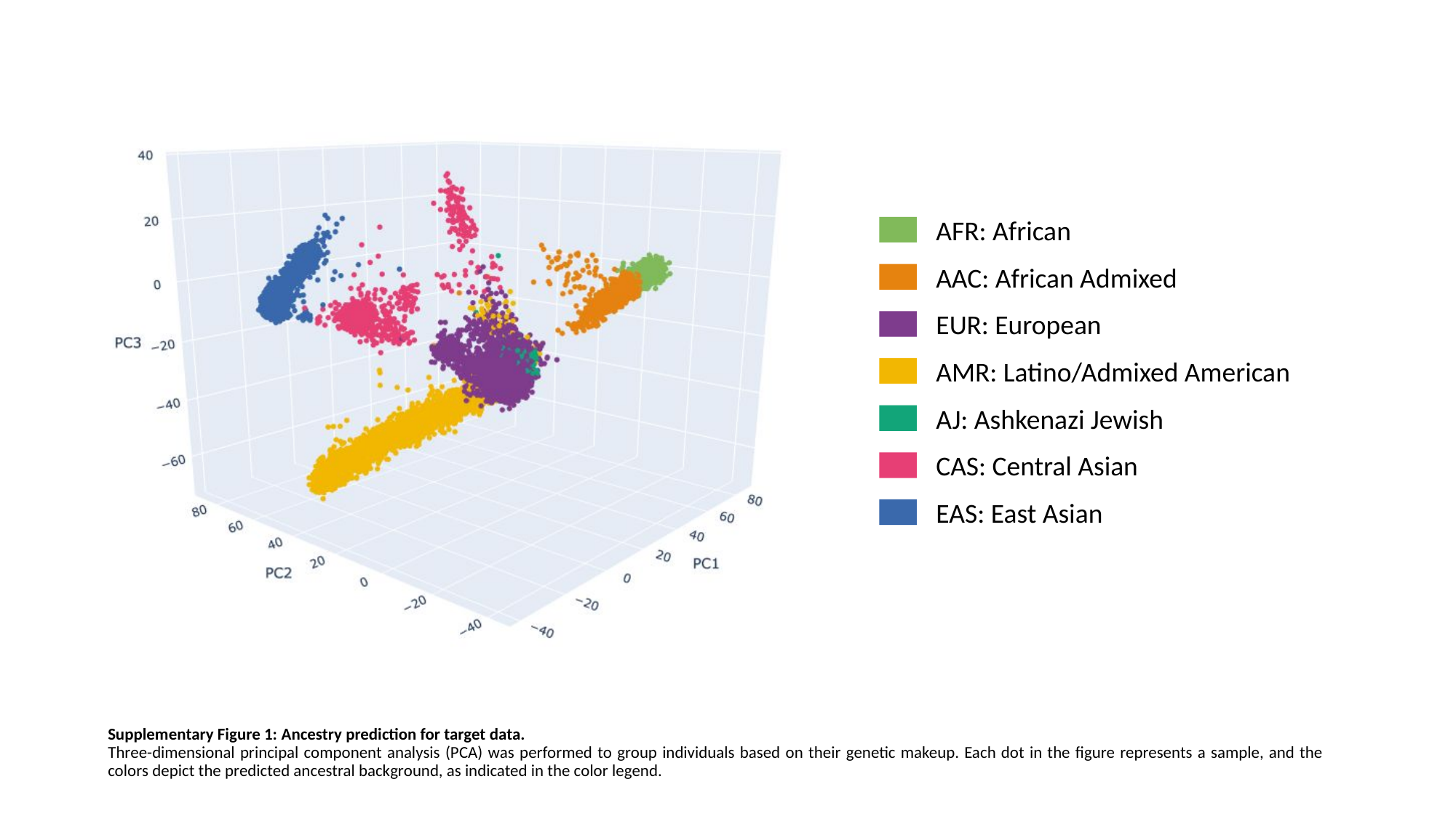

AFR: African
AAC: African Admixed
EUR: European
AMR: Latino/Admixed American
AJ: Ashkenazi Jewish
CAS: Central Asian
EAS: East Asian
Supplementary Figure 1: Ancestry prediction for target data.
Three-dimensional principal component analysis (PCA) was performed to group individuals based on their genetic makeup. Each dot in the figure represents a sample, and the colors depict the predicted ancestral background, as indicated in the color legend.

## Slide 2
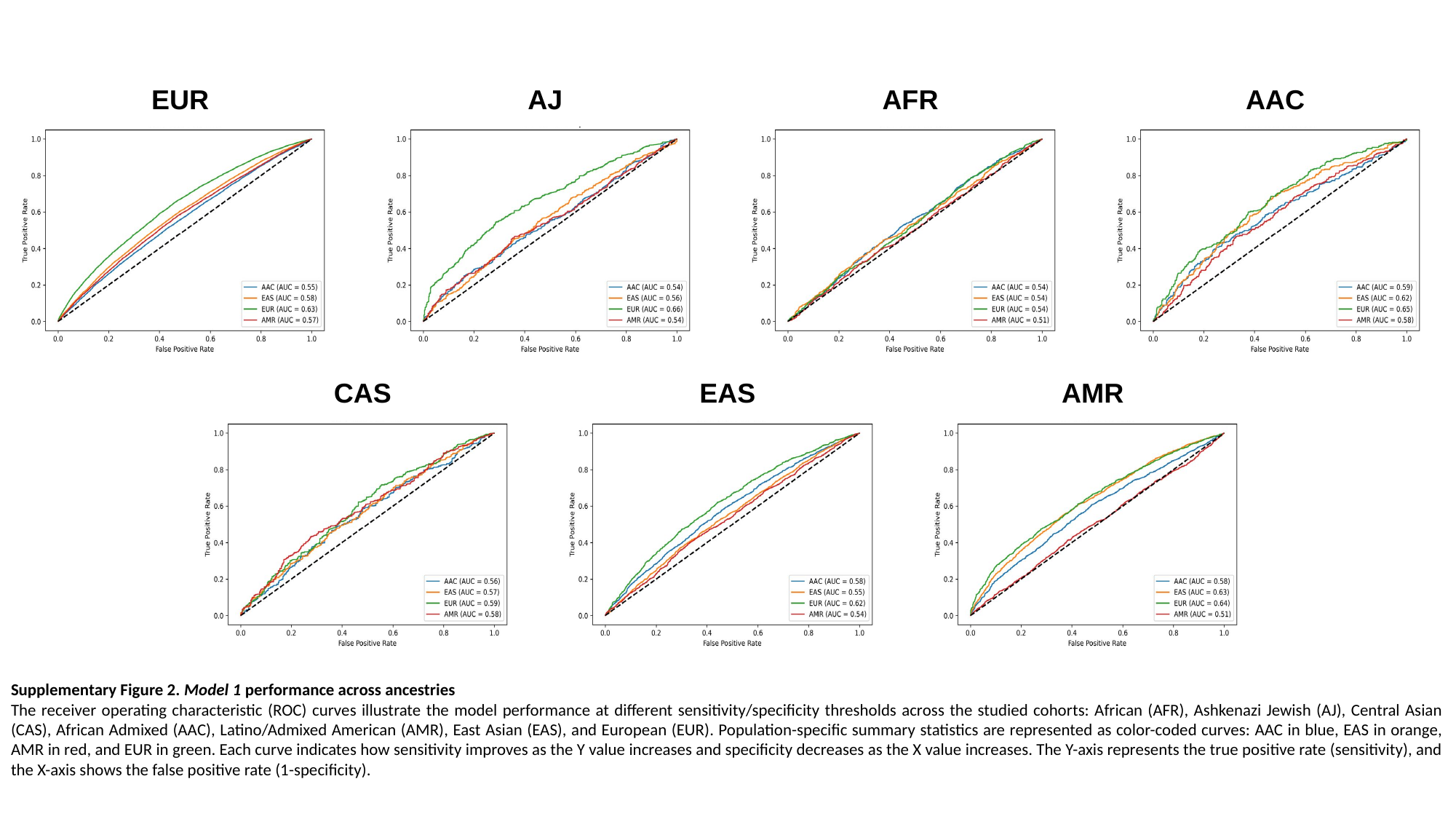

EUR
AJ
AFR
AAC
CAS
EAS
AMR
Supplementary Figure 2. Model 1 performance across ancestries
The receiver operating characteristic (ROC) curves illustrate the model performance at different sensitivity/specificity thresholds across the studied cohorts: African (AFR), Ashkenazi Jewish (AJ), Central Asian (CAS), African Admixed (AAC), Latino/Admixed American (AMR), East Asian (EAS), and European (EUR). Population-specific summary statistics are represented as color-coded curves: AAC in blue, EAS in orange, AMR in red, and EUR in green. Each curve indicates how sensitivity improves as the Y value increases and specificity decreases as the X value increases. The Y-axis represents the true positive rate (sensitivity), and the X-axis shows the false positive rate (1-specificity).

## Slide 3
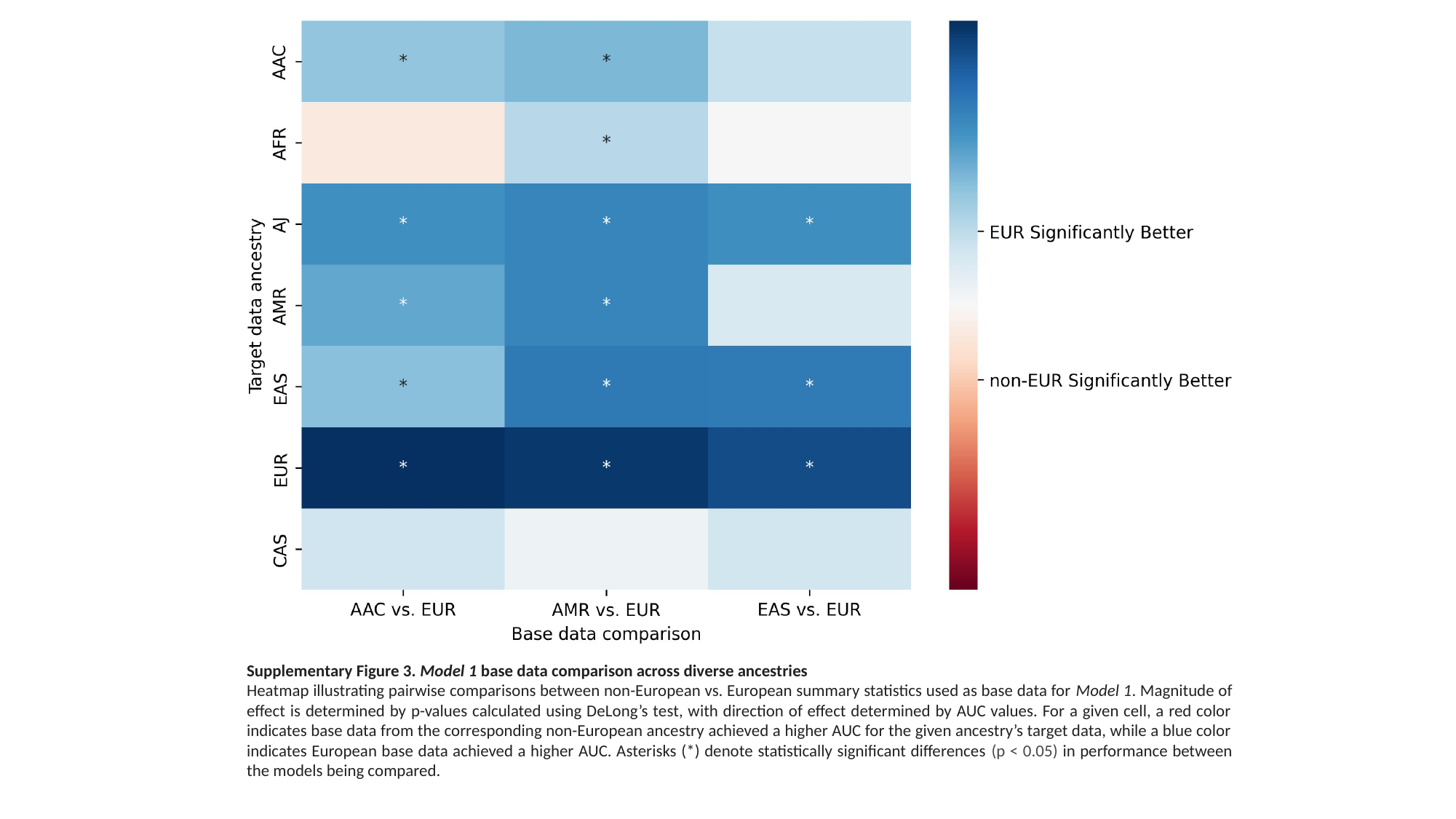

Supplementary Figure 3. Model 1 base data comparison across diverse ancestries
Heatmap illustrating pairwise comparisons between non-European vs. European summary statistics used as base data for Model 1. Magnitude of effect is determined by p-values calculated using DeLong’s test, with direction of effect determined by AUC values. For a given cell, a red color indicates base data from the corresponding non-European ancestry achieved a higher AUC for the given ancestry’s target data, while a blue color indicates European base data achieved a higher AUC. Asterisks (*) denote statistically significant differences (p < 0.05) in performance between the models being compared.

## Slide 4
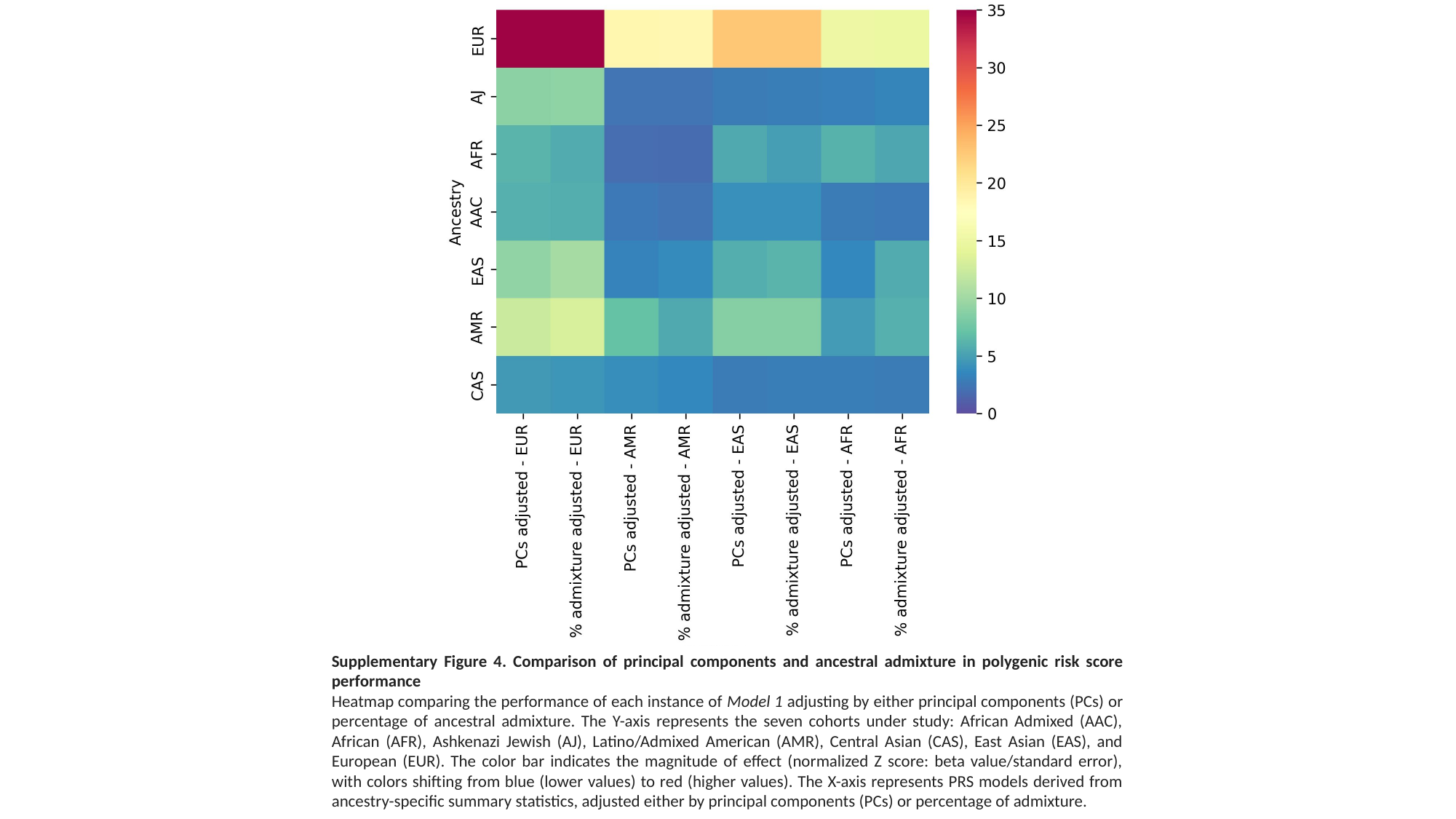

Supplementary Figure 4. Comparison of principal components and ancestral admixture in polygenic risk score performance
Heatmap comparing the performance of each instance of Model 1 adjusting by either principal components (PCs) or percentage of ancestral admixture. The Y-axis represents the seven cohorts under study: African Admixed (AAC), African (AFR), Ashkenazi Jewish (AJ), Latino/Admixed American (AMR), Central Asian (CAS), East Asian (EAS), and European (EUR). The color bar indicates the magnitude of effect (normalized Z score: beta value/standard error), with colors shifting from blue (lower values) to red (higher values). The X-axis represents PRS models derived from ancestry-specific summary statistics, adjusted either by principal components (PCs) or percentage of admixture.

## Slide 5
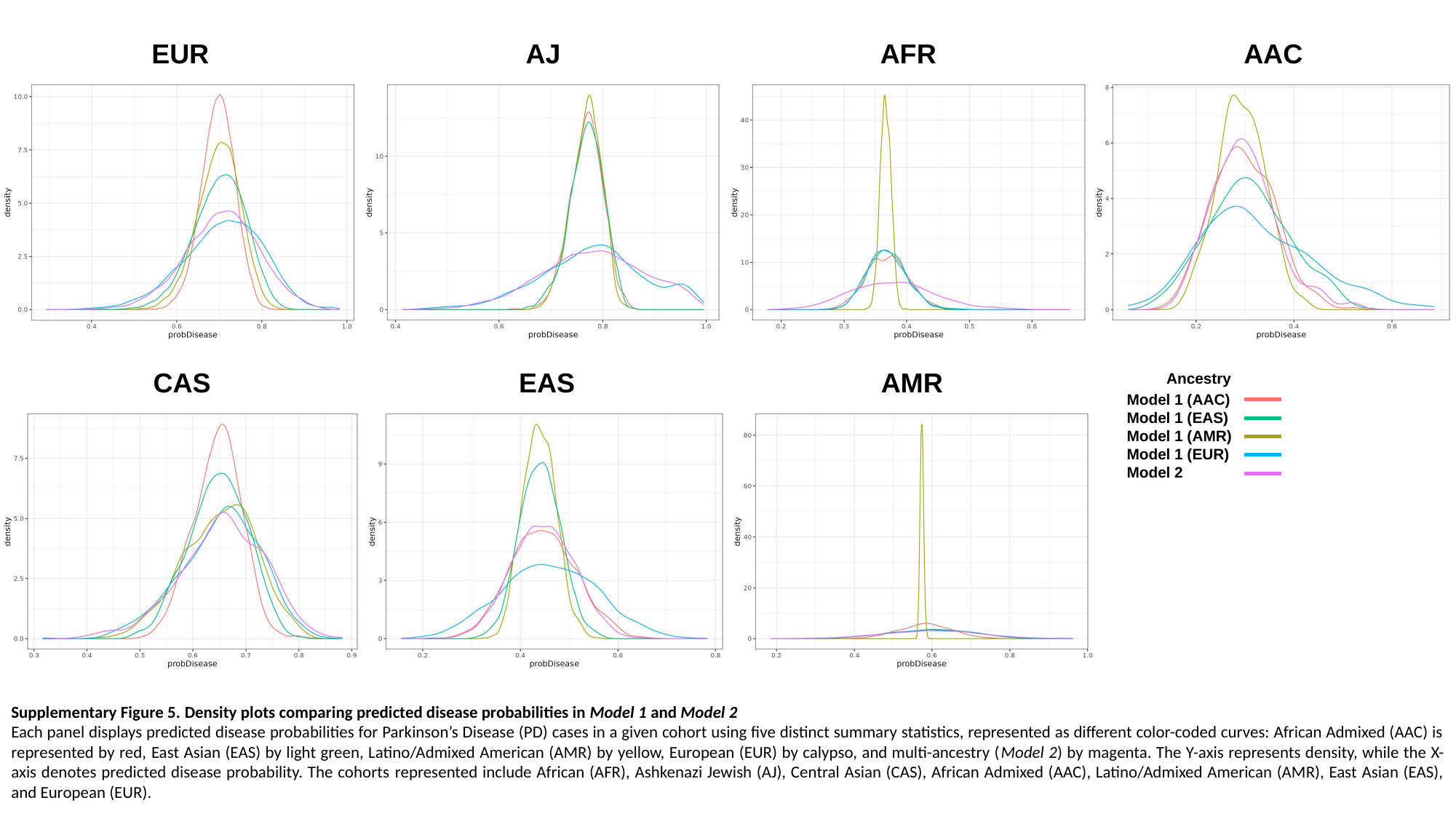

EUR
AJ
AFR
AAC
EAS
CAS
AMR
Ancestry
Model 1 (AAC)
Model 1 (EAS)
Model 1 (AMR)
Model 1 (EUR)
Model 2
Supplementary Figure 5. Density plots comparing predicted disease probabilities in Model 1 and Model 2
Each panel displays predicted disease probabilities for Parkinson’s Disease (PD) cases in a given cohort using five distinct summary statistics, represented as different color-coded curves: African Admixed (AAC) is represented by red, East Asian (EAS) by light green, Latino/Admixed American (AMR) by yellow, European (EUR) by calypso, and multi-ancestry (Model 2) by magenta. The Y-axis represents density, while the X-axis denotes predicted disease probability. The cohorts represented include African (AFR), Ashkenazi Jewish (AJ), Central Asian (CAS), African Admixed (AAC), Latino/Admixed American (AMR), East Asian (EAS), and European (EUR).

## Slide 6
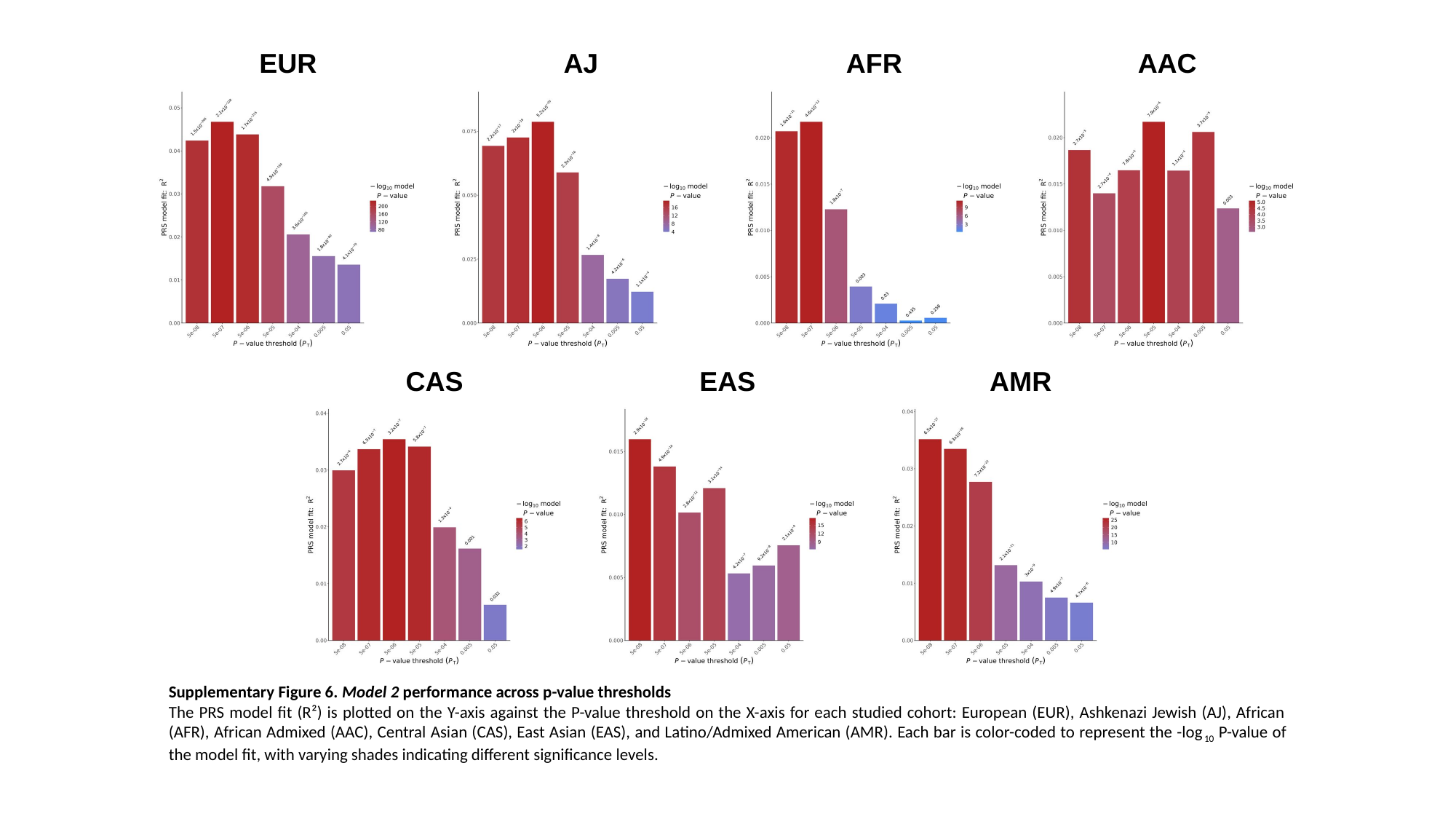

EUR
AJ
AFR
AAC
CAS
EAS
AMR
Supplementary Figure 6. Model 2 performance across p-value thresholds
The PRS model fit (R²) is plotted on the Y-axis against the P-value threshold on the X-axis for each studied cohort: European (EUR), Ashkenazi Jewish (AJ), African (AFR), African Admixed (AAC), Central Asian (CAS), East Asian (EAS), and Latino/Admixed American (AMR). Each bar is color-coded to represent the -log10 P-value of the model fit, with varying shades indicating different significance levels.
